# A Few Remarks on Asthma

**Published:** 1904-12

**Authors:** 


### A FEW REMARKS ON ASTHMA.

Asthma is entirely a spasmodic condition produced by a spasm or contraction of the circular muscular fibers of the air-tubes by which the tube caliber is reduced and breathing becomes abnormally difficult. It is a most oppressive condition and when the spasm is over it leaves the patient much exhausted. It is essential to prevent the asthmatic attack as far as it is possible. The point of greatest importance in the treatment of the sufferer is the improvement of the general health; if this can be accomplished the system is necessarily supplied with normal power to fight the cause or causes which bring on an attack and to stand the strain of the attack. A constant shortness of breath, aggravated at times by colds after exposure indicates either an asthmatical or emphysemic condition. In this condition the air-cells are abnormally dilated and frequently torn so that they coalesce one with another and the normal elasticity of the lung is greatly reduced, consequently, the patient can not properly empty the lungs. As the result of this the chest becomes barrel-shaped and the respiratory movements are very much diminished, and consequently the blood is very imperfectly oxygenated and the general system shows a malnutrition and anemia. To prevent the tearing of the cells into each other, as well as the spasms, it is highly necessary to improve the condition of the lung tissues by building up the general system. Where this is completely accomplished it relieves the distressing sensation or shortness of breath. It lessens the tendency to rupture and reduces the respiratory spasm. To restore the natural nutrition of the lung tissue is to enable it to recover its elasticity and this can only be done completely by supplying an absolute and perfect nutrition.

In a large clinical experience I have found that Bovinine meets every demand and can be given with impunity at all ages. It supplies perfect nutrition, tones up the enfeebled circulation—and keeps up a proper and gentle stimulation.—E. E. ROWELL, JR., M.D.

